# Impact of Low-Dose Computed Tomography Findings on Cigarette Smoking Cessation Among High-Risk Adults Participating in Lung Cancer Screening

**DOI:** 10.1093/ntr/ntaf010

**Published:** 2025-04-29

**Authors:** Evangelos Katsampouris, Amyn Bhamani, Fanta Bojang, Jennifer L Dickson, Helen Hall, Carolyn Horst, Priyam Verghese, Andrew Creamer, Ruth Prendecki, Chuen Khaw, Sophie Tisi, John McCabe, Kylie Gyertson, Anne-Marie Hacker, Laura Farrelly, Neal Navani, Allan Hackshaw, SUMMIT Consortium, Sam M Janes, Jennifer L Dickson, Carolyn Horst, Sophie Tisi, Helen Hall, Priyam Verghese, Andrew Creamer, Thomas Callender, Ruth Prendecki, Amyn Bhamani, Mamta Ruparel, Allan Hackshaw, Laura Farrelly, Jon Teague, Anne-Marie Mullin, Kitty Chan, Rachael Sarpong, Malavika Suresh, Samantha L Quaife, Arjun Nair, Anand Devaraj, Kylie Gyertson, Vicky Bowyer, Ethaar El-Emir, Judy Airebamen, Alice Cotton, Kaylene Phua, Elodie Murali, Simranjit Mehta, Janine Zylstra, Karen Parry-Billings, Columbus Ife, April Neville, Paul Robinson, Laura Green, Zahra Hanif, Helen Kiconco, Ricardo McEwen, Dominique Arancon, Nicholas Beech, Derya Ovayolu, Christine Hosein, Sylvia Patricia Enes, Qin April Neville, Jane Rowlands, Aashna Samson, Urja Patel, Fahmida Hoque, Hina Pervez, Sofia Nnorom, Moksud Miah, Julian McKee, Mark Clark, Jeannie Eng, Fanta Bojang, Claire Levermore, Anant Patel, Sara Lock, Rajesh Banka, Angshu Bhowmik, Ugo Ekeowa, Zaheer Mangera, William M Ricketts, Neal Navani, Terry O’Shaughnessy, Charlotte Cash, Magali Taylor, Samanjit Hare, Tunku Aziz, Stephen Ellis, Anthony Edey, Graham Robinson, Alberto Villanueva, Hasti Robbie, Elena Stefan, Charlie Sayer, Nick Screaton, Navinah Nundlall, Lyndsey Gallagher, Andrew Crossingham, Thea Buchan, Tanita Limani, Kate Gowers, Kate Davies, John McCabe, Joseph Jacob, Karen Sennett, Tania Anastasiadis, Andrew Perugia, James Rusius, Geoff Bellingan, Maureen Browne, Eleanor Hellier, Catherine Nestor, Sam M Janes, Samantha L Quaife, Sam M Janes, Jennifer L Dickson, Carolyn Horst, Sophie Tisi, Helen Hall, Priyam Verghese, Andrew Creamer, Thomas Callender, Ruth Prendecki, Amyn Bhamani, Mamta Ruparel, Allan Hackshaw, Laura Farrelly, Jon Teague, Anne-Marie Mullin, Kitty Chan, Rachael Sarpong, Malavika Suresh, Samantha L Quaife, Arjun Nair, Anand Devaraj, Kylie Gyertson, Vicky Bowyer, Ethaar El-Emir, Judy Airebamen, Alice Cotton, Kaylene Phua, Elodie Murali, Simranjit Mehta, Janine Zylstra, Karen Parry-Billings, Columbus Ife, April Neville, Paul Robinson, Laura Green, Zahra Hanif, Helen Kiconco, Ricardo McEwen, Dominique Arancon, Nicholas Beech, Derya Ovayolu, Christine Hosein, Sylvia Patricia Enes, Qin April Neville, Jane Rowlands, Aashna Samson, Urja Patel, Fahmida Hoque, Hina Pervez, Sofia Nnorom, Moksud Miah, Julian McKee, Mark Clark, Jeannie Eng, Fanta Bojang, Claire Levermore, Anant Patel, Sara Lock, Rajesh Banka, Angshu Bhowmik, Ugo Ekeowa, Zaheer Mangera, William M Ricketts, Neal Navani, Terry O’Shaughnessy, Charlotte Cash, Magali Taylor, Samanjit Hare, Tunku Aziz, Stephen Ellis, Anthony Edey, Graham Robinson, Alberto Villanueva, Hasti Robbie, Elena Stefan, Charlie Sayer, Nick Screaton, Navinah Nundlall, Lyndsey Gallagher, Andrew Crossingham, Thea Buchan, Tanita Limani, Kate Gowers, Kate Davies, John McCabe, Joseph Jacob, Karen Sennett, Tania Anastasiadis, Andrew Perugia, James Rusius, Geoff Bellingan, Maureen Browne, Eleanor Hellier, Catherine Nestor

**Affiliations:** Centre for Cancer Screening, Prevention, Detection and Early Diagnosis, Wolfson Institute of Population Health, Barts and The London School of Medicine and Dentistry, Queen Mary University of London, London, UK; Lungs for Living Research Centre, UCL Respiratory, University College London, London, UK; University College London Hospitals NHS Foundation Trust, London, UK; Lungs for Living Research Centre, UCL Respiratory, University College London, London, UK; Lungs for Living Research Centre, UCL Respiratory, University College London, London, UK; Lungs for Living Research Centre, UCL Respiratory, University College London, London, UK; Lungs for Living Research Centre, UCL Respiratory, University College London, London, UK; Lungs for Living Research Centre, UCL Respiratory, University College London, London, UK; Lungs for Living Research Centre, UCL Respiratory, University College London, London, UK; Lungs for Living Research Centre, UCL Respiratory, University College London, London, UK; Lungs for Living Research Centre, UCL Respiratory, University College London, London, UK; Lungs for Living Research Centre, UCL Respiratory, University College London, London, UK; University College London Hospitals NHS Foundation Trust, London, UK; Cancer Research UK and UCL Cancer Trials Centre, University College London, London, UK; Cancer Research UK and UCL Cancer Trials Centre, University College London, London, UK; Lungs for Living Research Centre, UCL Respiratory, University College London, London, UK; University College London Hospitals NHS Foundation Trust, London, UK; Cancer Research UK and UCL Cancer Trials Centre, University College London, London, UK; Lungs for Living Research Centre, UCL Respiratory, University College London, London, UK; Centre for Cancer Screening, Prevention, Detection and Early Diagnosis, Wolfson Institute of Population Health, Barts and The London School of Medicine and Dentistry, Queen Mary University of London, London, UK

## Abstract

**Introduction:**

Integrating effective smoking cessation strategies for individuals undergoing lung cancer screening stands to significantly increase the impact of lung screening programmes. We assessed the impact of low-dose computed tomography (LDCT) findings on smoking cessation among high-risk adults who currently smoked.

**Aims and Methods:**

13 035 individuals, aged 55–77 years, attended a lung health check appointment, as part of a prospective observational cohort study (the SUMMIT Study), prior to undergoing a baseline LDCT scan. Logistic regressions examined the likelihood of smoking cessation at a 1-year follow-up appointment and its association with LDCT findings.

**Results:**

12.6% (*n* = 647/5135) of individuals self-reported smoking cessation at 1-year follow-up. Higher odds of quitting were found in those receiving indeterminate pulmonary nodule findings requiring a 3-month interval LDCT (aOR = 1.27; 1.01, 1.61), those with urgent findings requiring referral to secondary care (aOR = 1.55; 1.05, 2.32), and those with a possible new chronic obstructive pulmonary disease diagnosis (aOR = 1.60; 1.23, 2.06), compared to those receiving no actionable LDCT findings. Older age, Asian ethnic background, current high smoking intensity, motivation and number of quit attempts, and low nicotine dependence were associated with increased odds of quitting.

**Conclusions:**

Individuals currently smoking, at high lung cancer risk, participating in LDCT screening, and receiving incidental findings requiring a 1-year interval LDCT or primary care follow-up might therefore need additional behavioral support to quit. Tailored communication strategies depending on the severity of the LDCT findings, including additional behavioral support for those with less clinical concerning or negative findings, could increase quit rates and reduce smoking-related morbidity.

**Implications:**

This study reports high odds of self-reported complete smoking cessation in adults who currently smoked after receiving their LDCT findings. Though the impact of specific types of LDCT findings on smoking cessation was positive for high lung cancer risk individuals, reception of incidental findings could potentially be perceived as less severe to encourage individuals who currently smoked to quit. Clearly communicating the severity of LDCT findings along with the delivery of behavioral smoking cessation support targeted to high-risk individuals may increase their chances of complete smoking cessation and reduce lung cancer mortality.

## Introduction

Lung cancer is the leading cause of cancer-related mortality worldwide.^1^ Tobacco smoking is the main risk factor for lung cancer in the United Kingdom (UK) and United States (US),^1,2^ causing an estimated 86% of cases.^3^ Smoking cessation remains the crucial preventive strategy for lung cancer among those who currently smoke.^4^ After smoking cessation, the most effective way to reduce lung cancer mortality for high-risk individuals is low-dose computed tomography (LDCT) screening for early-stage disease.^5,6^ A national lung cancer screening programme was recently recommended in the UK by the National Screening Committee,^7^ while national LDCT screening programmes have been implemented in the US, Taiwan, Poland, Croatia, South Korea, Czech Republic, and some regions of China.^8^

In the US, the primary risk-based eligibility criteria for LDCT screening consist of age (ie, 50–80 years) and a significant and recent tobacco smoking history (ie, typically a 20 pack-year smoking history and current smoking, or having quit within the past 15 years).^9^ In the UK, eligibility for lung cancer screening is based on validated risk assessment scores that consider risk factors including age (ie, 55–74 years), tobacco smoking history, personal or family history of lung cancer, personal history of respiratory illness, chronic obstructive pulmonary disease or emphysema, environmental and occupational factors, health-related quality of life, and body-mass index.^10,11^ Approximately half of participants in lung cancer screening studies currently smoke at the time of enrollment.^3,6,12^ Evidence suggests that the experience of lung cancer screening itself could increase motivation to quit and that the additive effect of smoking abstinence in the context of lung cancer screening increases the relative risk reduction in lung cancer mortality from lung cancer screening to 38%,^13–15^ resulting in substantial life-years gained and reductions in smoking-related morbidity and mortality.^16^

Participation in LDCT screening has been found to be associated with increased rates of smoking cessation compared to the general population.^15,17,18^ The Danish Lung Cancer Screening Trial (DLCST) found that approximately 12% of people who currently smoked in both arms had quit smoking at 1-year follow-up assessment.^19^ Additionally, in the UK Lung Cancer Screening (UKLS) trial 14% and 24% of individuals who currently smoked at baseline and underwent LDCT screening had quit smoking 2 weeks and up to 2 years since joining UKLS, respectively.^18^ However, the majority of lung cancer screening participants who smoke continue to do so following screening,^20^ reflecting the difficulty of overcoming long-term (and often life-long) tobacco dependence and the limited effectiveness of smoking cessation provision in the context of lung cancer screening to date. Investigating the factors associated with higher quit success is crucial for tailoring smoking cessation support appropriately and for capitalizing on the opportunity to provide effective support and maximizing the reduction in lung cancer mortality that lung cancer screening programmes can achieve.^21,22^

There has been significant interest in the impact of varying LDCT findings on smoking cessation but inconsistent evidence to date. Some studies suggest that abnormal LDCT findings are associated with higher quit rates compared to negative findings,^18,23–26^ while other studies found no substantial differences in smoking abstinence rates between individuals with abnormal and negative results.^27,28^ Increased smoking cessation rates have been reported after a finding that is immediately suspicious for lung cancer or a positive LDCT result compared to a negative finding.^23,24,29^ A recent study suggested that approximately 61.3% and 31.5% of participants in a residential smoking cessation programme continued to be abstinent from smoking 6 and 12 months, respectively, after their LDCT screening and that indeterminate or suspicious malignant lung nodule findings were associated with higher odds of 12-month continuous smoking abstinence.^30^ The odds of quitting have also been associated with demographic (eg, age, gender, ethnicity, socioeconomic deprivation) and smoking-related factors (eg, nicotine dependence, quit attempts, motivation to quit, smoking intensity), but again, their impact on smoking cessation has not been consistent among studies.^18,23,24,26,28^ To inform smoking cessation strategies and identify those who may need additional support, a better understanding is required of the factors affecting the likelihood of smoking cessation following LDCT screening.

The SUMMIT Study is a prospective observational cohort study that aims to assess the implementation of LDCT screening for lung cancer in a high-risk population in North Central and East London and validate a multi-cancer early detection blood test (NCT03934866). Here, we assess the proportion of individuals who currently smoked cigarettes at their baseline appointment, and subsequently reported having quit smoking completely at their 1-year follow-up appointment. We also report the extent to which demographic, smoking-related factors and LDCT findings at participants’ baseline Lung Health Check (LHC) appointment predicted the odds of complete smoking cessation at their 1-year follow-up appointment.

## Methods

### Participant Recruitment and Lung Health Check Appointments

Eligibility for participation in the SUMMIT Study was confirmed following attendance at a face-to-face baseline LHC. At this appointment, individuals who either met the 2013 United States Preventive Services Task Force LDCT screening criteria^9^ or had a ≥1.3% 6-year lung cancer risk based on the Prostate, Lung, Colorectal, and Ovarian model^31^ were invited to participate. The baseline LHC questionnaire included a self-reported assessment of lung cancer risk factors including smoking history and past medical history (including Chronic Obstructive Pulmonary Disease, COPD), and respiratory symptoms. Pre-bronchodilator spirometry was performed for individuals attending prior to the SARS-CoV-2 pandemic. Age and gender were obtained from primary care records and Index of Multiple Deprivation (IMD) ranks from residential postcodes. A baseline LDCT scan was carried out following this appointment.

Participants were invited to return for face-to-face LHC appointments at 1 (Year 1, between 13 July 2020 and 11 January 2023) and 2 (Year 2) year intervals, unless they had been diagnosed with lung cancer in the interim. At these appointments, they were asked about their smoking status and categorized as individuals who currently smoke or with a former smoking history.

### Measures

#### Cigarette Smoking History and Smoking Cessation

The baseline LHC included a detailed assessment of smoking history, including pack-year history, and smoking status (categorized into individuals who currently smoke or with smoking history) at the time of the appointment. Self-reported smoking status for those who currently smoke was determined by a “Yes” response to the question, “Do you currently smoke cigarettes regularly?.” Smoking cessation was determined at Year 1 assessment by a “Former smoker” response to the question, “Which of these options best describes your current smoking status?.” For analysis purposes, and aligning with previous research, only plausible values for cigarettes per day (CPD) and age started smoking were included in the analysis (ie, “Number of cigarettes or grams of tobacco”: 1–80 CPD or equivalent in grams of tobacco per week, and “At what age did you start smoking cigarettes regularly?”: ≥5 years).^32,33^

Individuals with current tobacco use (eg, cigarettes, pipes, cigars, cigarillos) at any study visit were offered Very Brief Advice (accredited by the National Center for Smoking Cessation and Training) on smoking cessation by a nurse or trial practitioner conducting the LHC appointment, and asked if they would consent to a referral to their free local stop smoking service being made on their behalf or to be signposted to self-refer to another stop smoking service if there was no local service.^34^

#### Smoking Behaviors

Upon completion of the baseline LHC, all individuals eligible for lung cancer screening who participated in the study were asked to complete an electronic questionnaire that collected further information on the level of nicotine dependence among participants who currently smoked, general health, lifestyle, and family history of lung cancer (ie, “Have your parents, brother, sister, or children ever have been diagnosed with lung cancer?”).

Measures of smoking behaviors included an assessment of motivation to quit using the Motivation to Stop Scale (MTSS),^35^ confidence in being able to quit among those reporting they were motivated to quit (“On a scale of 1–10, with 1 being the lowest and 10 being the highest, how confident are you that you will be able to stop smoking?”), the number of serious quit attempts made in the past 12 months (“How many serious attempts to stop smoking have you made in the last 12 months? By serious attempt we mean you decided that you would try to make sure you never smoked again. Please include any attempt that you are currently making.”), and time to first cigarette (TTFC) after waking as a measure of nicotine dependence (“How soon after you wake do you smoke your first cigarette?”).

MTSS responses were dichotomized, enabling comparison between those reporting a high level of motivation to quit in a defined time frame (ie, “I really want to stop smoking and intend to in the next 3 months” and “I really want to stop smoking and intend to in the next month”) and those reporting low motivation (ie, “I don’t want to stop smoking,” “I think I should stop smoking but don’t really want to,” “I want to stop smoking but haven’t thought about when,” “I really want to stop smoking but I don’t know when I will,” “I want to stop smoking and hope to soon”).^36^ The number of serious quit attempts in the past year (none, 1–4 attempts, ≥5 attempts), confidence to quit (low, moderate, high), and TTFC (within 5 minutes, 6–30 min, 31–60 min, >60 min) were analyzed categorically.^37^

#### LDCT and LHC Findings

Baseline LDCT and LHC findings were reported back to participants and their General Practitioner via postal letter. This included undiagnosed COPD in individuals with no self-reported history of this condition, airflow obstruction on spirometry (FEV_1_/FVC ratio < 0.7), and either a chronic productive cough or exertional dyspnea in keeping with a mMRC score ≥ 1.^38,39^ Participants with urgent LDCT findings also received a telephone call from a clinical member of the study team to discuss their results.

LHC and LDCT results were classified into the following categories:

1. Findings requiring urgent secondary care referral (suspected lung and non-lung cancer, and urgent non-cancer findings).
2. Indeterminate pulmonary nodules requiring 3-month interval LDCT within the study.
3. Incidental findings requiring 1-year interval LDCT within the study (<3cm anterior mediastinal masses, diffuse pleural thickening, ground glass opacities, and 1–4 cm adrenal nodules).
4. Incidental findings requiring primary care follow-up (including thyroid nodules, interstitial lung disease with fibrotic features, severe bronchiectasis)^40^.
5. Undiagnosed COPD only.
6. No actionable LDCT or LHC findings.

### Study Outcome Measures and Statistical Analysis

The primary objective of this analysis was to determine the proportion of individuals currently smoking cigarettes at their baseline LHC appointment who subsequently reported having a smoking history at their year 1 appointment. Secondary analyses tested for associations between individuals’ baseline demographic and smoking characteristics, their baseline LDCT findings, and the likelihood of cigarette smoking cessation 1 year after their baseline LHC appointment. All individuals who attended and completed a baseline LHC between 8 April 2019 and 14 May 2021 were included in the analysis; 74 individuals did not complete a baseline LDCT and did not attend subsequent study visits.

Logistic regression models were used to examine factors associated with smoking cessation 1 year after participants’ LHC appointment. Four demographic variables (gender, age, ethnicity, and IMD quintile); two cigarette smoking history variables (CPD and age started smoking); one family history of cancer variable; and four smoking behavior variables (TTFC, previous serious quit attempts, motivation to quit, and confidence to quit) were entered as predictor variables. Odds ratios are reported unadjusted and adjusted for the effects of other variables in the model.

Additionally, the LDCT finding categories variable was entered as a sole predictor variable, adjusted for the effects of gender, age, ethnicity, IMD quintile, CPD, TTFC, previous quit attempts, and motivation to quit. Odds ratios in these regression models indicate the proportionate change in participants’ odds of reporting cigarette smoking cessation 1 year after their LHC appointment associated with the indicator on the categorical predictor variable. Statistical analyses were carried out using IBM SPSS statistics (version 28.0.0). The *p* values < .05 were considered statistically significant.

## Results

### Participant Characteristics

About 13 035 individuals completed a baseline LHC between April 2019 and May 2021. Approximately half (48.9%, *n* = 6377/13 035) currently smoke cigarettes. Of those who currently smoked, 57.6% (*n* = 3673/6377) were male, and the mean age was 64.2 years (*SD* = 5.9, range = 55–79). Most (79.6%; *n* = 5075/6377) were of a White ethnic background and were living in areas categorized within the two most deprived national socioeconomic quintiles (65.8%; *n* = 4196/6377) (Table S1).

### Factors Associated with Cigarette Smoking Cessation 1 Year After the LHC and LDCT Screening Scan Appointment

80.5% (*n* = 5135/6377) of participants who reported currently smoking cigarettes at their baseline LHC subsequently attended a Y1 appointment. Self-reported smoking cessation rates among these individuals (ie, the efficacy sample) was 12.6% (*n* = 647/5135), and 10.1% for the intention-to-treat sample (*n* = 647/6377), at the 1-year follow-up assessment. Participants’ adjusted odds of reporting cigarette smoking cessation 1 year after their LHC appointment were significantly associated with demographic characteristics and behavioral factors (Table S2).

Participants aged 65–69 (aOR = 1.49; 1.16, 1.91) and 70–74 years (aOR = 1.60; 1.22, 2.11), and those of an Asian (aOR = 1.47; 1.08, 2.00) and any other (aOR = 1.83; 1.19, 2.81) ethnic background were more likely to have quit cigarette smoking 1 year after their LHC appointment relative to those aged 55–59 years and those of White ethnic background, respectively.

Compared to those who self-reported smoking less than 20 CPD, those who were smoking 60–79 CPD (aOR = 2.40; 1.07, 5.39) were more likely to have quit smoking. Compared to those who self-reported low levels of motivation to quit, those with high levels of motivation (aOR = 2.34; 1.90, 2.88) were more likely to have quit smoking.

Participants who made between 1 and 4 serious quit attempts in the last 12 months (relative to no attempts: aOR = 1.41; 1.18, 1.70) and those who self-reported smoking their first cigarette more than 60 min after waking (relative to within 5 min of waking: aOR = 2.49; 1.92, 3.24) were more likely to have quit cigarette smoking.

### Low-Dose Computed Tomography Findings at Baseline Associated With Cigarette Smoking Cessation 1 Year After LHC Appointment

Participants’ adjusted odds of reporting cigarette smoking cessation 1 year after their LHC appointment significantly varied by their LDCT findings at baseline (Table 1).

**Table 1.** Logistic Regression Models of the Association Between Baseline Low-Dose Computed Tomography Findings and Cigarette Smoking Cessation 1 Year After the Baseline Lung Health Check Appointment

Predictor variable	% SC-Y1	Unadjusted OR (95% CI)	aOR (95% CI)^a^
LDCT finding categories	*n* = 5135		*n* = 4932
No actionable LDCT or LHC findings (*n* = 319/2860)	11.2	Ref.	Ref.
Indeterminate pulmonary nodule findings requiring 3-month interval LDCT (*n* = 118/883)	13.4	1.23 (0.98–1.54)	1.27 (1.01–1.61)^*^
Findings requiring urgent secondary care referral (*n* = 35/200)	17.5	1.69 (1.15–2.48)^**^	1.55 (1.04–2.32)^*^
Incidental findings requiring 1-year interval LDCT (*n* = 56/429)	13.1	1.20 (0.88–1.62)	1.10 (0.79–1.52)
Incidental findings requiring primary care follow-up (*n* = 22/174)	12.6	1.15 (0.73–1.83)	1.11 (0.68–1.80)
Undiagnosed COPD only (*n* = 97/589)	16.5	1.57 (1.23–2.01)^***^	1.60 (1.23–2.06)^***^

SC, smoking cessation; Y1, 1 year after Lung Health check appointment; OR, odds ratio; CI, confidence interval; LDCT, low-dose computed tomography;

^*^ *p* < .05;

^**^ *p* < .01; and

^***^ *p* ≤ .001.

^a^aORs were adjusted for the effects of gender, age, ethnicity, national index of multiple deprivation rank quintile, cigarettes per day (CPD), motivation to quit, quit attempts, and nicotine dependence.

Compared to those who had no actionable LDCT or LHC findings, those with indeterminate pulmonary nodule findings requiring 3-month interval LDCT (aOR = 1.27; 1.01, 1.61), those with urgent findings requiring referral to secondary care (aOR = 1.55; 1.05, 2.32) and those with a possible new diagnosis of COPD that was reported back to their GP (aOR = 1.60; 1.23, 2.06) had 27%, 55%, and 60% higher odds, respectively, of having quit cigarette smoking 1 year after their LHC appointment.

## Discussion

This study prospectively assessed factors associated with cigarette smoking quit rates in a large cohort of adults participating in a multisite implementation trial of lung cancer screening in socioeconomically and ethnically diverse areas of London. Factors associated with increased likelihood of smoking cessation at 1 year were indeterminate pulmonary nodule findings requiring 3-month interval LDCT, urgent findings requiring referral to secondary care, or a possible new diagnosis of COPD. Additionally, individuals who were relatively older (aged 65–74 years) and those of an Asian or other ethnic background were more likely to have quit, as were those who smoked a higher number of cigarettes daily (between 60 and 79 cigarettes), had high motivation to quit, had made several (between 1 and 4) attempts to quit the year before their first scan, and had low nicotine dependence.

Our findings contribute further evidence for the impact of specific types of abnormal LDCT results on smoking cessation outcomes. Observational evidence of smoking cessation among individuals participating in controlled trials of LDCT screening has shown that the impact of LDCT lung screening on cigarette smoking cessation was positive for individuals who received a positive LDCT screening result (including abnormal results requiring referral to a multidisciplinary team and incidental findings) compared to those with negative findings.^18,23^ Unlike previous evidence, no associations were found between incidental findings (eg, adrenal nodules, thyroid nodules, severe bronchiectasis) and smoking cessation.^18^ This could be explained in this study that COPD was analyzed separately from other incidental findings and thus it could have been that driving the association with smoking cessation found in other studies.^41^ Indeterminate or suspicious lung nodule results have also been found to be associated with a higher 12-month continuous smoking cessation in a residential smoking cessation programme compared to a 6-month assessment.^30^ Interestingly, the present study reported that those with indeterminate results and those with urgent findings requiring referral to secondary care had a higher likelihood of having quit at 1-year assessment, although we did not assess continuous abstinence rates. More quit attempts have also been observed to be associated with those receiving an indeterminate result,^28^ as in the present study which found that those who made several quit attempts were more likely to quit in adjusted analyses suggesting a significant association between quit attempts and smoking cessation.

Similar to previous studies, compared to those with no actionable LDCT findings, those with indeterminate pulmonary nodule findings were more likely to have quit smoking 12 months after their LHC appointment. The present study also found self-reported smoking cessation rates of 12.6% at 1-year follow-up assessment among individuals currently smoking at a baseline LHC appointment. This quit rate is lower than those from the UKLS and ITALUNG studies (24% in the LDCT screening group after approximately 2 years and 20.8% in the LDCT screening group up to 4 years follow-up, respectively), which reported that people currently smoking who received an abnormal finding (eg, result requiring referral to the multidisciplinary team, incidental findings, findings suspicious for lung cancer) at baseline had a higher likelihood to quit smoking, although the follow-up timescale assessments crucially vary among the studies.^18,42^ However, the present quit rate is similar to other lung screening studies (11.9% in the DLCST and 12.1% in the Mayo study after 1 year of screening, and 13.1% for the LDCT screening group in the 2-year follow-up assessment in the NELSON),^19,24,43,44^ which could be explained by the fact that among individuals who currently smoke in these studies self-reported that they were highly motivated to stop smoking. Embedding LDCT screening with smoking cessation interventions could prompt quitting and encourage sustained abstinence among motivated individuals who currently smoke, as this has also been highlighted in other LCS programmes.^18,43–45^ Evidence from the Yorkshire Enhanced Stop Smoking study (YESS), delivered within the Yorkshire Lung Screening Trial, has shown considerably higher quit rates, 28.6% in the control group (receiving standard best practice smoking cessation support) and 29.2% in the intervention group (receiving best practice smoking and a personalized booklet with LDCT images), respectively, at 12 months after the screening visit. This supports the provision of a more intensive co-located smoking cessation approach, over and above that offered by the SUMMIT Study, to better support those entering lung screening with long-term tobacco dependence.^46^ Lastly, it was important to understand the smoking behavior among those not currently smoking when attending lung screening, who might have experienced a relapse and returned to regular smoking.

Contrary to previous evidence that demonstrated no statistically significant association between the type of LDCT finding and smoking cessation in males,^15,27,28^ the present study suggests that LDCT lung cancer screening increases smoking cessation among those who received either indeterminate findings, urgent findings, or a possible new diagnosis of COPD, regardless of gender. We also present evidence for differences by ethnic group for the first time in the UK lung cancer screening context, finding that Asian ethnicity was associated with higher reported smoking cessation. This is supported by data from NHS Stop Smoking Services in England showing that those of an Asian ethnic background make more attempts to quit smoking and to set a quit date than those from any other ethnic group (7 vs. 3, respectively).^47^ However, the majority of our sociodemographic findings largely align with previous evidence that those of relatively older age, with higher motivation to quit and with lower nicotine dependency are more likely to quit smoking.^19,23–25,44,48^ The UKLS trial deviates from this picture slightly, showing that older age was not significantly associated with either short or long-term smoking cessation,^18^ although in the present study, those in the oldest category (75–79 years) were not more likely to quit in adjusted analyses suggesting the association with age may not be linear.

Although the present study found that those who self-reported the heaviest smoking intensity (over 60 cigarettes daily) were more likely to quit, this should be treated with caution due to the small number of cases in this group, and the opposite direction of association for increasing smoking intensity for the other groups (ie, odds of quitting decreased as reported consumption increased except in this higher group). It is also not supported by existing evidence, which has shown that heavy smoking intensity was associated with a high risk of continued smoking.^23^ Those who participate in lung cancer screening might be more confident and have high intention to and the likelihood of quitting as participation in lung cancer screening might be seen as a “teachable moment.”^21^ As in the UKLS trial,^18^ no significant associations between socioeconomic deprivation levels (ie, IMD rank) and smoking cessation were found. However, the broader literature shows a consistent socioeconomic gradient in smoking cessation,^23,49,50^ despite individuals experiencing deprivation making the same number of quit attempts.^51^ It is possible that this is a self-selection effect, where those from lower socioeconomic groups able to attend lung cancer screening are also those with more opportunity and motivation to quit, although the present analyses were adjusted for quit motivation and nicotine dependency. Future research should seek to test this finding in other lung cancer screening settings and explore in greater depth how smoking cessation interventions might need to be tailored according to characteristics like deprivation, especially considering that the cigarette smoking prevalence is highest in the most deprived areas.

The limitations of this study are acknowledged. This was an observational study with only two timepoints to assess the impact of receiving LDCT findings and negative scan results on cigarette SC among high-risk adults participating in lung cancer screening. Smoking cessation was measured 1 year after the baseline LHC appointment and there might have existed other factors (eg, duration of smoking abstinence) that could affect the outcome. Assessment of smoking history, including duration and average consumption, and current smoking status was based on a collection of individuals’ self-reported data; this study would have further benefitted by collecting CO-validated smoking readings though in this context we regard this as a minor concern. Although this study found that over 1 in 10 people who currently smoked quit 12 months after a baseline LHC appointment, it is not known what proportion of those remained abstinent from smoking in the intervening period nor the longer term. We suggest that qualitative research is needed to better understand individuals’ approaches to LDCT results’ severity, for example, whether people currently smoking perceive incidental findings or a possible new COPD diagnosis as less severe compared to abnormal nodule findings, which subsequently might affect smoking cessation. This study assessed associations between demographic and behavioral characteristics and LDCT findings on smoking cessation; the impact of confidence to quit would have also been assessed if self-reported baseline data was complete. Last, future research should examine the quit rates among those who received a brief information intervention and were offered the opportunity for a referral to free smoking cessation services.

In conclusion, this prospective observational study highlighted the impact of specific types of abnormal LDCT results on cigarette smoking cessation among high-risk adults following an LHC appointment. This study suggests that receiving indeterminate pulmonary nodule findings requiring 3-month interval LDCT, urgent findings requiring referral to secondary care, or a possible new diagnosis of COPD increases the likelihood of successfully quitting smoking, when incidental results (requiring either 1-year interval LDCT or primary care follow-up) do not. Relatively older age, an Asian ethnic background, higher number of cigarettes smoked per day, high motivation and high number of serious attempts to quit, and low nicotine dependence might constitute characteristics among individuals currently smoking who have higher odds of quitting cigarette smoking. Tailoring communication practices for the severity of LDCT findings to individuals at high risk of developing lung cancer and prioritizing the delivery of appropriate tobacco dependence treatment is likely to result in greater quit and abstinence rates from cigarette smoking in the longer term and thus in reduced smoking-related morbidity and mortality.

SUMMIT consortium members

Sam M. Janes¹, Jennifer L. Dickson¹, Carolyn Horst¹, Sophie Tisi¹, Helen Hall¹, Priyam Verghese¹, Andrew Creamer¹, Thomas Callender¹, Ruth Prendecki¹, Amyn Bhamani¹, Mamta Ruparel¹, Allan Hackshaw², Laura Farrelly², Jon Teague², Anne-Marie Mullin², Kitty Chan², Rachael Sarpong², Malavika Suresh², Samantha L. Quaife³, Arjun Nair^4^, Anand Devaraj⁵,⁶, Kylie Gyertson^4^, Vicky Bowyer^4^, Ethaar El-Emir ^4^, Judy Airebamen^4^, Alice Cotton^4^, Kaylene Phua^4^, Elodie Murali^4^, Simranjit Mehta^4^, Janine Zylstra^4^, Karen Parry-Billings^4^, Columbus Ife^4^, April Neville^4^, Paul Robinson^4^, Laura Green^4^, Zahra Hanif^4^, Helen Kiconco^4^, Ricardo McEwen^4^, Dominique Arancon^4^, Nicholas Beech^4^, Derya Ovayolu^4^, Christine Hosein^4^, Sylvia Patricia Enes^4^, Qin April Neville^4^, Jane Rowlands^4^, Aashna Samson^4^, Urja Patel^4^, Fahmida Hoque^4^, Hina Pervez^4^, Sofia Nnorom^4^, Moksud Miah^4^, Julian McKee^4^, Mark Clark^4^, Jeannie Eng^4^, Fanta Bojang^4^, Claire Levermore^4^, Anant Patel^7^, Sara Lock⁸, Rajesh Banka⁹, Angshu Bhowmik¹⁰, Ugo Ekeowa¹¹, Zaheer Mangera¹², William M Ricketts¹³, Neal Navani^4^, Terry O’Shaughnessy¹³, Charlotte Cash^7^, Magali Taylor^4^, Samanjit Hare^7^, Tunku Aziz¹³, Stephen Ellis¹³, Anthony Edey¹^4^, Graham Robinson¹⁵, Alberto Villanueva¹⁶, Hasti Robbie¹^7^, Elena Stefan¹⁸, Charlie Sayer¹⁹, Nick Screaton²⁰, Navinah Nundlall^4^, Lyndsey Gallagher^4^, Andrew Crossingham^4^, Thea Buchan^4^, Tanita Limani^4^, Kate Gowers¹, Kate Davies¹, John McCabe¹, Joseph Jacob¹ ²^4^, Karen Sennett²¹, Tania Anastasiadis ²², Andrew Perugia²³, James Rusius²³, Geoff Bellingan^4^, Maureen Browne^4^, Eleanor Hellier^4^, Catherine Nestor^4^

1. Lungs For Living Research Centre, UCL Respiratory, University College London, London; 2. CRUK & UCL Cancer Trials Centre, University College London, London; 3. Centre for Prevention, Detection and Diagnosis, Wolfson Institute of Population Health, Barts and The London School of Medicine and Dentistry, Queen Mary University of London, London; 4. University College London Hospitals NHS Foundation Trust, London; 5. Royal Brompton and Harefield NHS Foundation Trust, London; 6. National Heart and Lung Institute, Imperial College, London; 7. Royal Free London NHS Foundation Trust, London; 8. Whittington Health NHS Trust, London; 9. Barking, Havering and Redbridge University Hospitals NHS Trust, Essex; 10. Homerton University Hospital Foundation Trust, London; 11. The Princess Alexandra Hospital NHS Trust, Essex; 12. North Middlesex University Hospital NHS Trust, London; 13. Barts Health NHS Trust, London; 14. North Bristol NHS Trust, Bristol; 15. Royal United Hospitals Bath NHS Foundation Trust, Bath; 16. Surrey and Sussex Healthcare NHS Trust, Surrey; 17. King’s College Hospital NHS Foundation Trust, London; 18. The Princess Alexandra Hospital NHS Trust, London; 19. University Hospitals Sussex NHS Foundation Trust, Sussex; 20. Royal Papworth Hospital NHS Foundation Trust, Cambridge; 21. Killick Street Health Centre, London; 22. Tower Hamlets Clinical Commissioning Group, London; 23. Noclor Research Support, London; 24. Centre for Medical Image Computing, London. We kindly request that the collaborators are tagged for PubMed purposes.

## Supplementary Material

Supplementary material is available at *Nicotine and Tobacco Research* online.

ntaf010_suppl_Supplementary_Tables_1-2

## Data Availability

The data underlying this article will be shared on reasonable request to the corresponding author.
