# Supplementary material for: Impact of Low-Dose Computed Tomography Findings on Cigarette Smoking Cessation Among High-Risk Adults Participating in Lung Cancer Screening
Source: Nicotine Tob Res. 2025 Apr 29;27(8):1412–9. doi: 10.1093/ntr/ntaf010 (PMC12280174; doi:10.1093/ntr/ntaf010)
Supplement: ntaf010_suppl_Supplementary_Tables_1-2 [file ntaf010_suppl_supplementary_tables_1-2.docx]

**Supplementary Table 1.** Characteristics of SUMMIT Study participants who reported currently smoking cigarettes at baseline (n = 6,377)

| **Variable** | **Frequency (n)** | **Percentage (%)** |
| --- | --- | --- |
| **Gender** |  |  |
| Male | 3,673 | 57.6 |
| Female | 2,704 | 42.4 |
| **Mean age (SD)** |  |  |
|  | 64.2 (5.9) | |
| **Age groups (years)** |  |  |
| 55-59 | 1,662 | 26.1 |
| 60-64 | 1,820 | 28.5 |
| 65-69 | 1,552 | 24.3 |
| 70-74 | 977 | 15.3 |
| 75-79 | 366 | 5.7 |
| **Ethnicity^1^** |  |  |
| White | 5,075 | 79.6 |
| Asian | 545 | 8.5 |
| Black | 374 | 5.9 |
| Mixed | 162 | 2.5 |
| Other | 221 | 3.5 |
| **National Index of Multiple Deprivation (IMD) rank** |  |  |
| Quintile 1 (most deprived) | 2,244 | 35.2 |
| Quintile 2 | 1,952 | 30.6 |
| Quintile 3 | 1,048 | 16.4 |
| Quintile 4 | 811 | 12.7 |
| Quintile 5 (least deprived) | 257 | 4.0 |
| Missing | 65 | 1.0 |
| **Cigarettes per day (CPD)** |  |  |
| < 20 | 2,928 | 45.9 |
| 20-39 | 3,130 | 49.1 |
| 40-59 | 253 | 4.0 |
| 60-79 | 46 | 0.7 |
| Missing | 20 | 0.3 |
| **Age started smoking (years)** | | |
| 6-10 | 246 | 3.9 |
| 11-20 | 5,320 | 83.4 |
| 21-30 | 731 | 11.5 |
| 31-40 | 61 | 1.0 |
| 41-50 | 10 | 0.2 |
| 51-60 | 4 | 0.1 |
| Missing | 5 | 0.1 |
| **Family history of lung cancer** | | |
| Yes | 1,185 | 18.6 |
| No | 5,192 | 81.4 |
| **Motivation to quit based on MTSS^2^** | | |
| Low | 5,432 | 85.2 |
| High | 869 | 13.6 |
| Missing | 76 | 1.2 |
| **Number of serious quit attempts to stop smoking in the last 12 months** | | |
| None | 3,697 | 58.0 |
| 1-4 | 2,050 | 32.1 |
| > 5 | 433 | 6.8 |
| Missing | 197 | 3.1 |
| **Time to first cigarette (TTFC)** | | |
| Within 5 minutes | 1,462 | 22.9 |
| 6-30 minutes | 2,583 | 40.5 |
| 31-60 minutes | 1,119 | 17.5 |
| > 60 minutes | 1,137 | 17.8 |
| Missing | 76 | 1.2 |
| **LDCT findings** | | |
| No actionable LDCT or LHC findings | 3,574 | 56.0 |
| Indeterminate pulmonary nodule findings requiring 3-month interval LDCT | 1,001 | 15.7 |
| Findings requiring urgent secondary care referral | 331 | 5.2 |
| Incidental findings requiring 1-year interval LDCT | 459 | 7.2 |
| Incidental findings requiring primary care follow-up | 218 | 3.4 |
| Undiagnosed COPD only | 752 | 11.8 |
| Missing | 42 | 0.7 |

Note: ^1^ White: British, Irish, Other; Mixed: White and Black Caribbean, White and Black African, White and Asian, Other; Asian: Chinese, Indian, Pakistani, Bangladeshi, Other; Black: Black Caribbean, Black African, Other

^2^ Low: I don’t want to stop smoking, I think I should stop smoking but really don’t want to, I want to stop smoking but I haven’t thought about when, I really want to stop smoking but I don’t know when I will, I want to stop smoking and hope to soon; High: I really want to stop smoking and intend to in the next three months, I really want to stop smoking and intend to in the next month

**Supplementary Table 2.** Frequencies and associations with smoking cessation one year after the baseline Lung Health Check appointment

|  | **Frequency within each demographic or smoking subgroup who reported quitting smoking at year 1 Lung Health Check** | | **Associations with quitting smoking at year 1 Lung Health Check** | |
| --- | --- | --- | --- | --- |
|  | **N** | **Percentage (%)** | **Unadjusted OR (95% CI)** | **Adjusted OR (95% CI)**  **n = 4,928** |
| **Gender** |  | | |  |
| Male (n = 2,982) | 382 | 12.8 | Ref. | Ref. |
| Female (n = 2,153) | 265 | 12.3 | 0.96 (0.81-1.13) | 0.98 (0.82-1.17) |
| **Age groups (years)** |  | | |  |
| 55-59 (n = 1,357) | 137 | 10.1 | Ref. | Ref. |
| 60-64 (n = 1,499) | 168 | 11.2 | 1.12 (0.89-1.43) | 1.12 (0.87-1.43) |
| 65-69 (n = 1,222) | 183 | 15.0 | 1.57 (1.24-1.99)*** | 1.49 (1.16-1.91)** |
| 70-74 (n = 783) | 120 | 15.3 | 1.61 (1.24-2.10)*** | 1.60 (1.22-2.11)*** |
| 75-79 (n = 274) | 39 | 14.2 | 1.48 (1.01-2.17)* | 1.33 (0.88-1.99) |
| **Ethnicity^1^** |  | | |  |
| White (n = 4,149) | 500 | 12.1 | Ref. | Ref. |
| Asian (n = 398) | 62 | 15.6 | 1.35 (1.01-1.79)* | 1.47 (1.08-2.00)* |
| Black (n = 281) | 30 | 10.7 | 0.87 (0.59-1.29) | 0.87 (0.58-1.31) |
| Mixed (n = 139) | 25 | 18.0 | 1.60 (1.03-2.49)* | 1.57 (0.98-2.50) |
| Other (n = 168) | 30 | 17.9 | 1.59 (1.06-2.38)* | 1.83 (1.19-2.81)** |
| **National Index of Multiple Deprivation (IMD) rank** |  | | |  |
| Quintile 1 (most deprived) (n = 1,799) | 227 | 12.6 | 0.90 (0.59-1.35) | 1.01 (0.65-1.55) |
| Quintile 2 (n = 1,529) | 173 | 11.3 | 0.79 (0.52-1.20) | 0.84 (0.54-1.30) |
| Quintile 3 (n = 858) | 113 | 13.2 | 0.94 (0.61-1.45) | 0.93 (0.59-1.46) |
| Quintile 4 (n = 680) | 100 | 14.7 | 1.07 (0.69-1.66) | 1.11 (0.70-1.76) |
| Quintile 5 (least deprived) (n = 216) | 30 | 13.9 | Ref. | Ref. |
| **Cigarettes per day (CPD)** |  | | |  |
| < 20 (n = 2,366) | 315 | 13.3 | Ref. | Ref. |
| 20-39 (n = 2,513) | 306 | 12.2 | 0.90 (0.76-1.07) | 1.08 (0.90-1.29) |
| 40-59 (n = 203) | 16 | 7.9 | 0.56 (0.33-0.94)* | 0.65 (0.37-1.15) |
| 60-79 (n = 37) | 9 | 24.3 | 2.09 (0.98-4.48) | 2.40 (1.07-5.39)* |
| **Age started smoking (years)** |  | | |  |
| 6-10 (n = 201) | 23 | 11.4 | Ref. | Ref. |
| 11-20 (n = 4,312) | 545 | 12.6 | 1.13 (0.72-1.75) | 0.96 (0.60-1.53) |
| 21-30 (n = 562) | 75 | 13.3 | 1.20 (0.73-1.97) | 0.92 (0.55-1.56) |
| 31-40 (n = 48) | 2 | 4.2 | 0.34 (0.08-1.49) | 0.23 (0.05-1.03) |
| 41-50 (n = 5) | 2 | 40.0 | 5.19 (0.82-32.71) | 6.41 (0.84-48.86) |
| 51-60 (n = 3) | 0 | - | - | - |
| **Family history of lung cancer** |  | | |  |
| Yes (n = 125) | 125 | 13.1 | Ref. | Ref. |
| No (n = 522) | 522 | 12.5 | 0.95 (0.77-1.17) | 0.86 (0.69-1.07) |
| **Motivation to quit based on MTSS^2^** |  | | |  |
| Low (n = 4,385) | 477 | 10.9 | Ref. | Ref. |
| High (n = 708) | 165 | 23.3 | 2.49 (2.04-3.04)*** | 2.34 (1.90-2.88)*** |
| **Number of serious quit attempts to stop smoking in the last 12 months** |  | | |  |
| None (n = 3,026) | 318 | 10.5 | Ref. | Ref. |
| 1-4 (n = 1,637) | 264 | 16.1 | 1.64 (1.37-1.95)*** | 1.41 (1.18-1.70)*** |
| > 5 (n = 338) | 50 | 14.8 | 1.48 (1.07-2.04)* | 1.23 (0.88-1.71) |
| **Time to first cigarette (TTFC)** |  | | |  |
| Within 5 minutes (n = 1,158) | 110 | 9.5 | Ref. | Ref. |
| 6-30 minutes (n = 2,093) | 214 | 10.2 | 1.09 (0.85-1.38) | 1.07 (0.83-1.37) |
| 31-60 minutes (n = 911) | 108 | 11.9 | 1.28 (0.97-1.70) | 1.21 (0.91-1.62) |
| > 60 minutes (n = 931) | 210 | 22.6 | 2.78 (2.16-3.56)*** | 2.49 (1.92-3.24)*** |

Note: OR = odds ratio; CI = confidence interval; *** p < 0.001, ** p < 0.01; * p < 0.05

^1^ White: British, Irish, Other; Mixed: White and Black Caribbean, White and Black African, White and Asian, Other; Asian: Chinese, Indian, Pakistani, Bangladeshi, Other; Black: Black Caribbean, Black African, Other

^2^ Low: I don’t want to stop smoking, I think I should stop smoking but really don’t want to, I want to stop smoking but I haven’t thought about when, I really want to stop smoking but I don’t know when I will, I want to stop smoking and hope to soon; High: I really want to stop smoking and intend to in the next three months, I really want to stop smoking and intend to in the next month
